# Bioinformatics and experimental analyses of glutamate receptor and its targets genes in myocardial and cerebral ischemia

**DOI:** 10.1186/s12864-023-09408-z

**Published:** 2023-06-02

**Authors:** Wei Liao, Chunming He, Shaochun Yang, Man Zhou, Chuan Zeng, Muyun Luo, Junjian Yu, Shuo Hu, Yanyu Duan, Ziyou Liu

**Affiliations:** 1 Medical College of Soochow University, Suzhou, Jiangsu China; 2 Department of Neurosurgery, First Affiliated of Gannan Medical University, Ganzhou, Jiangxi China; 3 Key Laboratory of Prevention and Treatment of Cardiovascular and Cerebrovascular Diseases, Ministry of Education, Gannan Medical University, Ganzhou, Jiangxi China; 4 Department of Cardiac Surgery, First Affiliated of Gannan Medical University, Ganzhou, Jiangxi China; 5 Heart Medical Centre, First Affiliated of Gannan Medical University, Ganzhou, Jiangxi China; 6 Gannan Medical University, Ganzhou, Jiangxi China

**Keywords:** Glutamate receptor, Myocardial ischemia, Ischemic stroke, Bioinformatics analysis, Combinational conservation of heart and brain

## Abstract

**Background:**

There is a mutual hemodynamic and pathophysiological basis between the heart and brain. Glutamate (GLU) signaling plays an important role in the process of myocardial ischemia (MI) and ischemic stroke (IS). To further explore the common protective mechanism after cardiac and cerebral ischemic injuries, the relationship between GLU receptor-related genes and MI and IS were analyzed.

**Results:**

A total of 25 crosstalk genes were identified, which were mainly enriched in the Toll-like receptor signaling pathway, Th17 cell differentiation, and other signaling pathways. Protein-protein interaction analysis suggested that the top six genes with the most interactions with shared genes were IL6, TLR4, IL1B, SRC, TLR2, and CCL2. Immune infiltration analysis suggested that immune cells such as myeloid-derived suppressor cells and monocytes were highly expressed in the MI and IS data. Memory B cells and Th17 cells were expressed at low levels in the MI and IS data; molecular interaction network construction suggested that genes such as JUN, FOS, and PPARA were shared genes and transcription factors; FCGR2A was a shared gene of MI and IS as well as an immune gene. Least absolute shrinkage and selection operator logistic regression analysis identified nine hub genes: IL1B, FOS, JUN, FCGR2A, IL6, AKT1, DRD4, GLUD2, and SRC. Receiver operating characteristic analysis revealed that the area under the curve of these hub genes was > 65% in MI and IS for all seven genes except IL6 and DRD4. Furthermore, clinical blood samples and cellular models showed that the expression of relevant hub genes was consistent with the bioinformatics analysis.

**Conclusions:**

In this study, we found that the GLU receptor-related genes IL1B, FOS, JUN, FCGR2A, and SRC were expressed in MI and IS with the same trend, which can be used to predict the occurrence of cardiac and cerebral ischemic diseases and provide reliable biomarkers to further explore the co-protective mechanism after cardiac and cerebral ischemic injury.

**Supplementary Information:**

The online version contains supplementary material available at 10.1186/s12864-023-09408-z.

## Introduction

Cardiac and cerebral damage caused by ischemic-hypoxic-reperfusion injury from cardiopulmonary resuscitation, severe compound injuries, and complex cardiovascular diseases are the major causes of acute mortality and chronic disability in patients [1]. This type of injury is an extremely complex pathophysiological cascade response process that includes a variety of damage repair mechanisms, such as neuronal autophagy, apoptosis, inflammatory damage, mitochondrial damage, and intracellular calcium overload [2–4]. Many studies have been conducted in the last decade on the treatment of ischemia-reperfusion injury in the heart and brain. Many studies have been conducted on the treatment of ischemic-reperfusion injury; for example, measures to enhance the perfusion, reduce oxygen consumption, and prevent ischemic-hypoxic damage to cardiac and cerebral cells have been used for the prevention and treatment of perioperative ischemic-hypoxic injury. However, there has been no fundamental improvement in prognosis after cardiac arrest. It is important to find effective preventive and therapeutic measures to prevent the damage caused by ischemic-hypoxia-reperfusion injury. Although there are significant functional and structural differences between the heart and brain, studies have found striking similarities in the mechanisms of injury between them, such as a common pathology: atherosclerosis [5] and the finding that most of the molecular events that occur after neurological injury are also present after myocardial injury [6], suggesting a common mechanism of injury between these two organs. The discovery of such mechanisms will provide new ideas for the complete and thorough elucidation of the molecular mechanisms of cardiac and cerebral injury and more effective protective measures. Therefore, it is an urgent and complex research topic in the field of organ protection to strengthen the joint protection of the heart and brain and to explore targets and measures for the prevention and treatment of ischemia-hypoxia-reperfusion injury in these two organs.

Many studies have confirmed that one of the mechanisms of cerebral ischemia-hypoxia-reperfusion injury is glutamate (GLU) excitotoxicity [7, 8], and that GLU receptors (GLuR) play an important role as major excitatory neurotransmitters in the central nervous system during ischemic stroke (IS). After the onset of ischemia and hypoxia, extracellular fluid GLU rises significantly, activating many GLuR and leading to inward calcium flow, resulting in GLU excitotoxicity and death signal activation. GLuR are also found in peripheral tissues such as the heart, pancreas, and bone [9–12]. We have previously reported that N-methyl-D-aspartate receptor can induce apoptosis of cardiomyocytes through the p38MAPK signaling pathway after myocardial ischemia (MI) and hypoxia. The GLuR blocker MK-801 not only showed a significant protective effect on the CA1 region of the rabbit hippocampus but also a significant reduction in cardiomyocyte apoptosis and a significant decrease in troponin I in a rabbit cardiac arrest cardiopulmonary resuscitation model, demonstrating a significant effect of cardio-cerebral protection. Moreover, it was found to be a signal transduction mechanism in MI-reperfusion injury [13–15]. These studies demonstrate that GLU signaling plays an important role in both cerebral and MI-hypoxia-reperfusion injuries.

The present study aimed to analyze the target genes and biological processes related to MI, IS, and GLuR using bioinformatics to identify common injury mechanisms and targets for heart and brain ischemia-hypoxia-reperfusion injury, to intervene and treat cardio-cerebral ischemia-hypoxia-reperfusion injury at an early stage, and to reduce its lethality and disability.

## Results

### Project flow chart

**Fig. 1:**
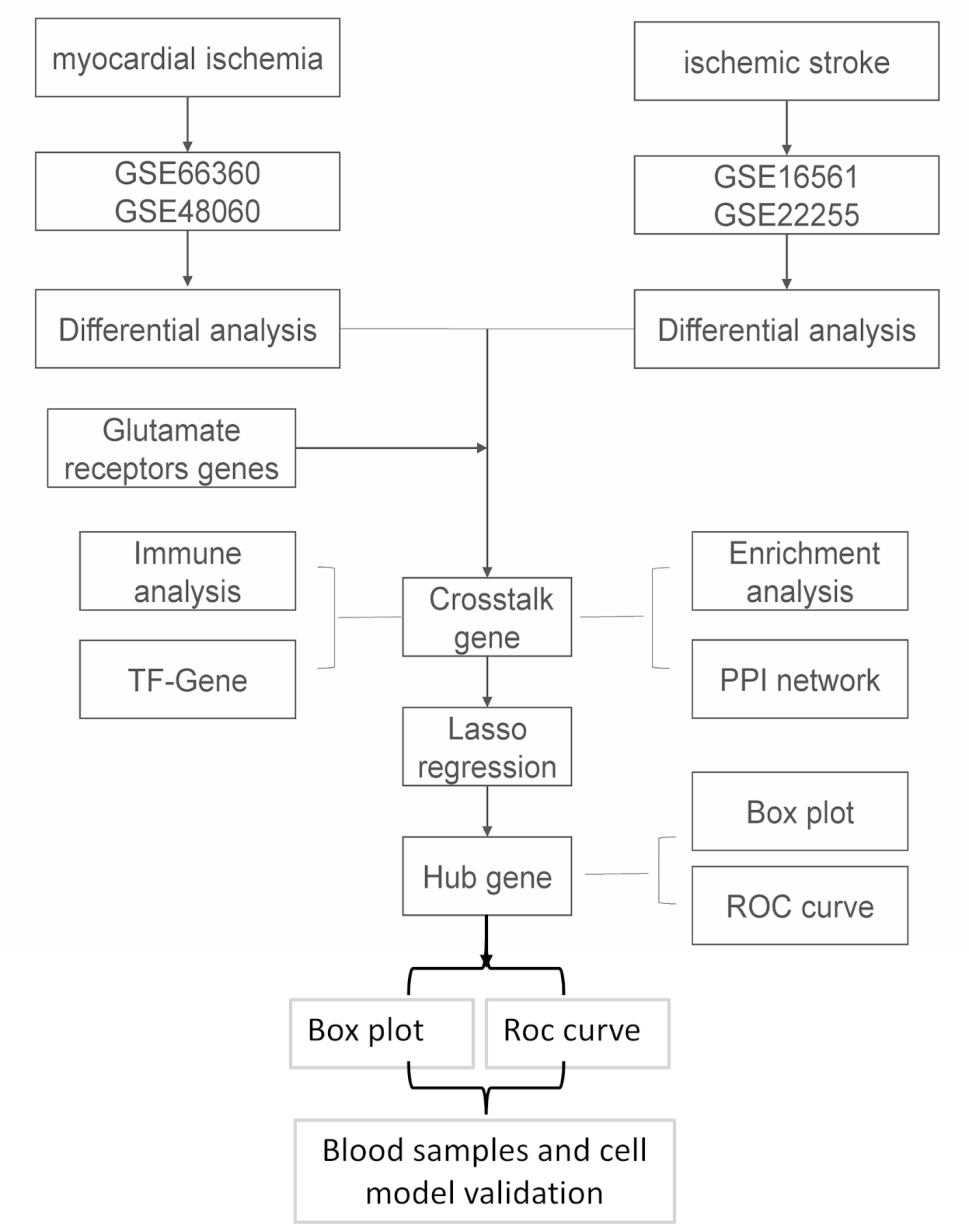
Flow chart of overall project analysis

In the current study, we analyzed the MI-related datasets GSE66360 and GSE48060, and the IS-related datasets GSE16561 and GSE22255. The differentially expressed genes (DEGs) were intersected with GLuR genes, and the crosstalk genes were subjected to reciprocal network construction, immune infiltration analysis, intersection gene-transcription factor (TF), pathway intersection gene/immune gene reciprocal network construction, and least absolute shrinkage and selection operator (LASSO) model construction. Finally, the key GLuR-related genes in MI and IS were obtained and validated by clinical specimens and cellular models (Fig. 1).

### DEG analysis

**Fig. 2:**
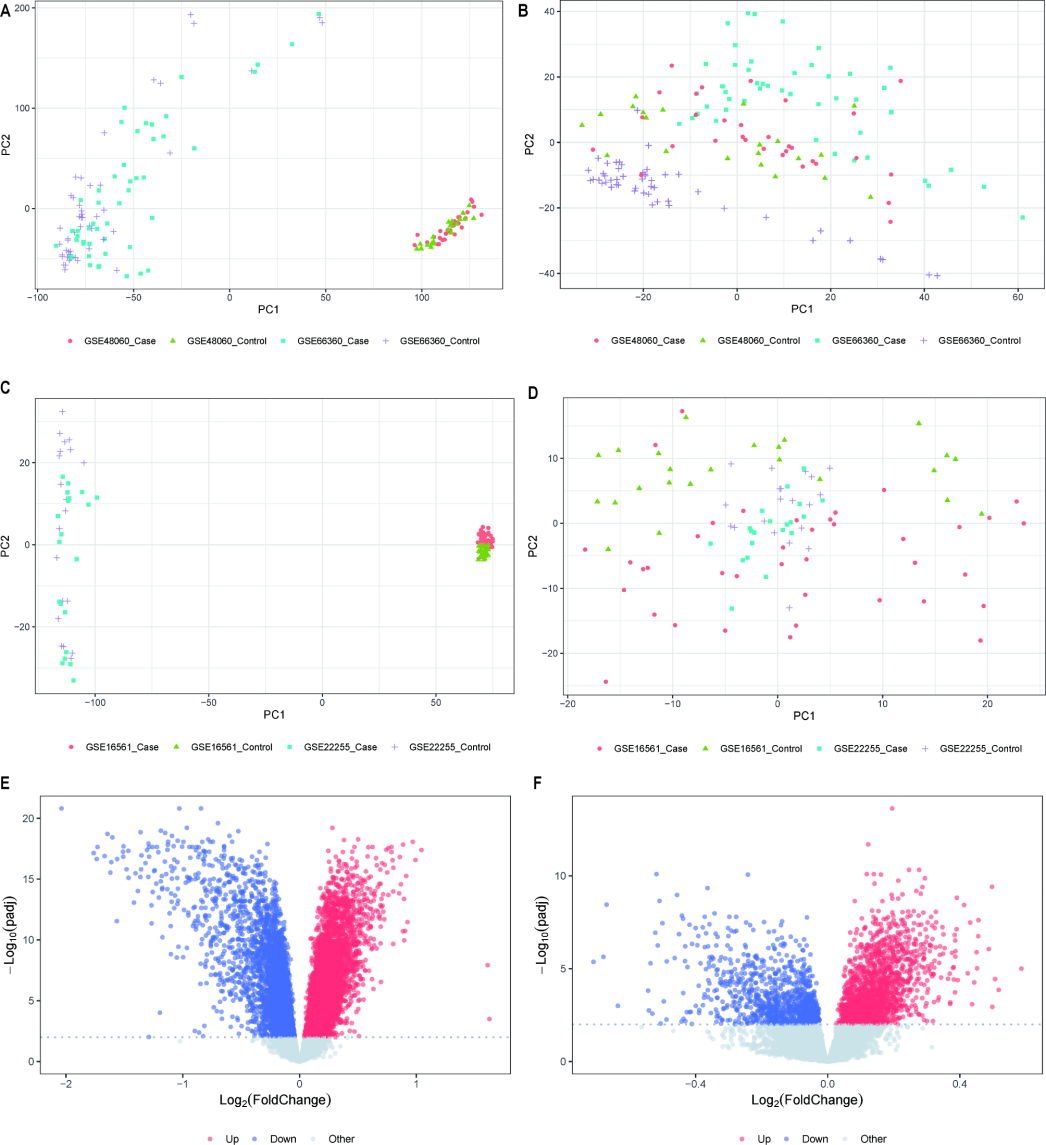
Data pre-processing and differential expression analysis. **(A)** PCA results before correction of gene expression profiles in MI data. (**B)** PCA results after correction of gene expression profiles in MI data. (**C)** PCA results before correction of gene expression profiles in IS data. (**D)** PCA results after correction of gene expression profiles in IS data. (**E)** MI expression profile data case vs. control differential expression volcano plot, horizontal coordinate is log2FoldChange, vertical coordinate is -log10 (p.adjust). Red nodes indicate upregulated DEGs, blue nodes indicate downregulated DEGs, and grey nodes indicate genes that are not significantly differentially expressed. (**F)** IS expression profile data case vs. control differential expression volcano plot, horizontal coordinate is log2FoldChange, vertical coordinate is -log10 (p.adjust), red nodes indicate upregulated DEGs, blue nodes indicate downregulated DEGs, and grey nodes indicate genes that are not significantly differentially expressed

The expression profiles of GSE66360 and GSE48060 from the MI-related dataset were combined after debatching, and PCA was performed on the expression values of the pre-corrected samples (Fig. 2A). The differences between the corrected samples were reduced (Fig. 2B). The expression profiles of GSE16561 and GSE22255 from the IS-related dataset were also debated, and PCA was performed on the expression values of the pre-corrected samples (Fig. 2C), whereas the differences between the post-corrected samples were reduced (Fig. 2D; Table 1).

**Table 1:** GEO data summary

	Series	Platforms	Case	Control	Total
MI	GSE66360	GPL570	49	50	99
	GSE48060	GPL570	31	21	52
IS	GSE16561	GPL6883	39	24	63
	GSE22255	GPL570	20	20	40

To analyze the effect of gene expression values on MI and IS samples relative to normal samples, we obtained DEGs for both datasets using the limma package differential analysis (Table 2) and created volcano plots against DEGs. A total of 10,602 DEGs were obtained from the MI data, including 5039 upregulated and 5563 downregulated genes (Fig. 2E). In addition, 2789 DEGs were identified from the IS data, including 1659 upregulated and 1130 downregulated genes (Fig. 2F).

**Table 2:** Summary of differential gene information

Disease	Up	Down	Total
MI	5039	5563	10,602
IS	1659	1130	2789

### Identification of intersecting genes

**Fig. 3:**
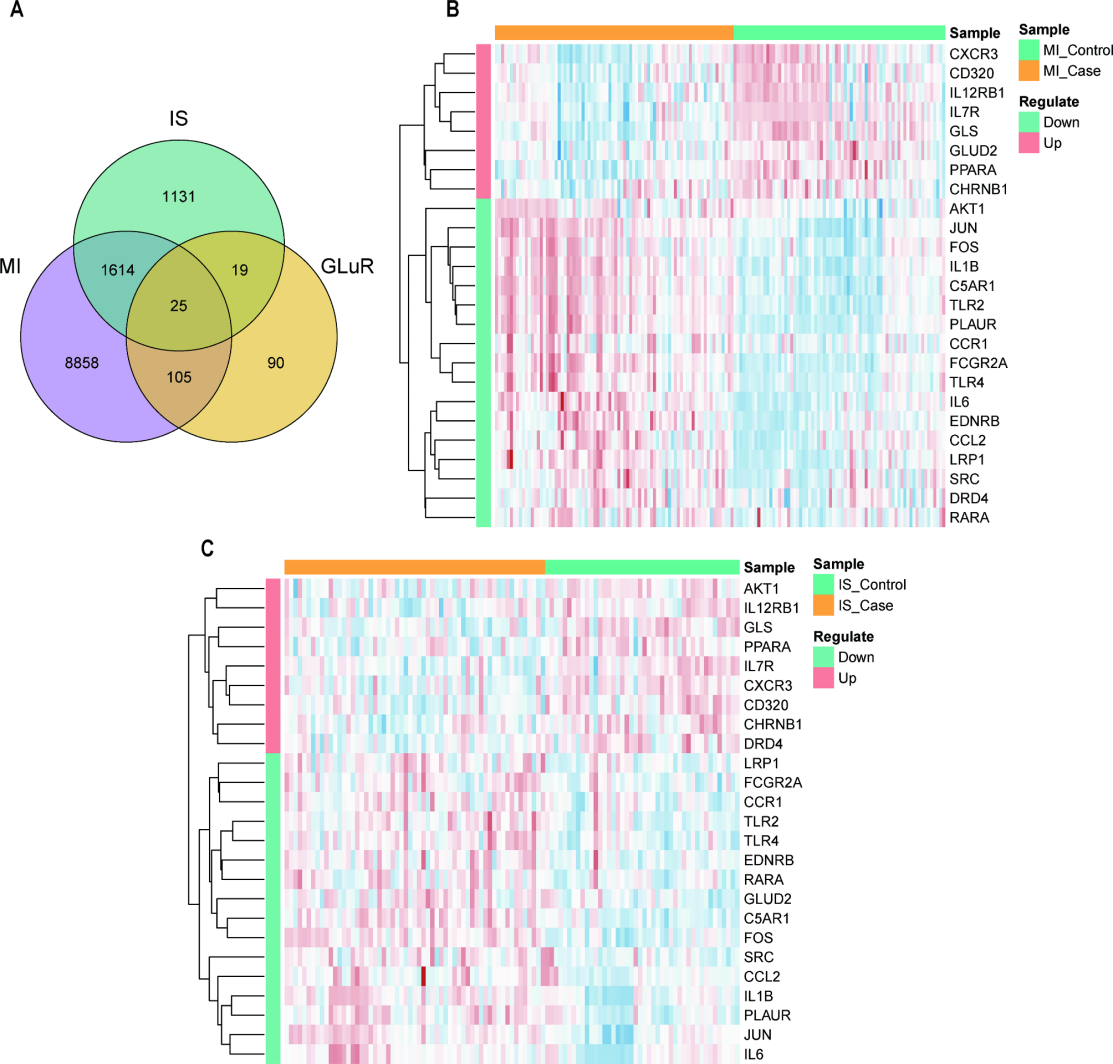
Identification of intersecting genes. (**A**) Wayne diagram of intersecting genes; blue circles are IS DEGs, purple circles are MI DEGs, yellow circles are GLuR-related genes. (**B**) Heat map of expression of intersecting genes in MI. (**C**) Heat map of intersecting gene expression in IS. IS: ischemic stroke; MI: myocardial ischemia; GLuR: glutamate receptor

To investigate the interaction between MI and IS, we examined the intersections of DEGs and GLuR-related genes from the MI and IS data to obtain 25 intersecting genes and plotted Venn diagrams (Fig. 3A). To investigate the types of these intersecting genes and their expression in different samples, we used the pheatmap package to analyze the expression of genes in the MI-related- and IS-related datasets (Fig. 3B and C). In the MI-related dataset, the 25 intersecting genes contained nine upregulated and 16 downregulated genes. In the IS dataset, the 25 intersecting genes contained eight upregulated and 17 downregulated genes. Among the 25 intersecting genes, the expression trends were the same in the MI and IS datasets, except for AKT1, DRD4, and GLUD2, which showed opposite trends.

### Functional enrichment analysis of intersecting genes

**Fig. 4:**
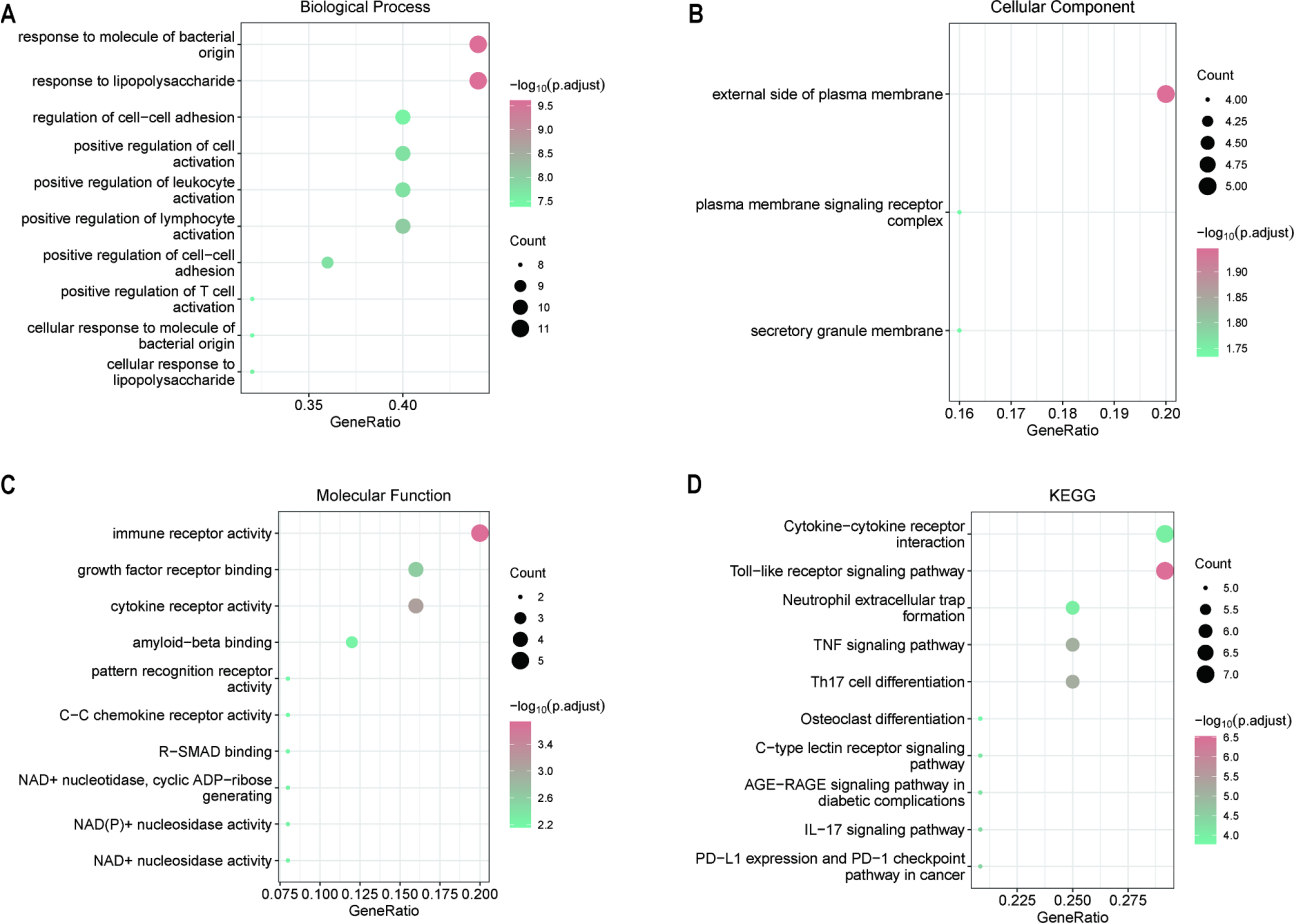
GO and KEGG enrichment analysis. Enrichment results for (**A**) biological process, (**B**) cellular component, (**C**) molecular function, and (**D**) KEGG pathway. For all graphs, y-axes are top 10 GO terms, node size indicates number of genes enriched in pathway, and node color indicates -log10 (p.adjust)

To analyze the relationship between the biological processes, molecular functions, cellular components, biological pathways, and diseases of the crossover genes, we performed gene ontology (GO) and Kyoto Encyclopedia of Genes and Genomes (KEGG) functional enrichment analyses of the crossover genes (Fig. 4; Tables 3 and 4). The GO functional enrichment analysis revealed that the crossover genes were mainly enriched in responses to lipopolysaccharide and molecules of bacterial origin, positive regulation of lymphocyte and leukocyte activation, cell-cell adhesion, cell activation, and other biological processes (Fig. 4A). The genes were also enriched in the external side of the plasma membrane, secretory granule membrane, plasma membrane signaling receptor complex cellular fractions (Fig. 4B), immune receptor activity, cytokine receptor activity, growth factor receptor binding, amyloid-beta binding, NAD + nucleosidase activity, NAD(P) + nucleosidase activity, and other molecular functions (Fig. 4C; Table 3). Pathway enrichment analysis of the intersecting genes showed enrichment in the Toll-like receptor (TLR) signaling pathway, Th17 cell differentiation, TNF signaling pathway, PD-L1 expression, PD-1 checkpoint pathway in cancer, IL17 signaling pathway, AGE-RAGE signaling pathway in diabetic complications, and other KEGG pathways (Fig. 4D; Table 4).

**Table 3:** GO enrichment analysis

ONTOLOGY	ID	Description	p.adjust
BP	GO:0032496	response to lipopolysaccharide	2.48E-10
BP	GO:0002237	response to molecule of bacterial origin	2.48E-10
BP	GO:0051251	positive regulation of lymphocyte activation	8.88E-09
BP	GO:0002696	positive regulation of leukocyte activation	1.86E-08
BP	GO:0022409	positive regulation of cell-cell adhesion	1.86E-08
BP	GO:0050867	positive regulation of cell activation	1.87E-08
BP	GO:0022407	regulation of cell-cell adhesion	2.85E-08
BP	GO:0071222	cellular response to lipopolysaccharide	2.95E-08
BP	GO:0071219	cellular response to molecule of bacterial origin	4.14E-08
BP	GO:0050870	positive regulation of T cell activation	4.14E-08
CC	GO:0009897	external side of plasma membrane	0.011341482
CC	GO:0030667	secretory granule membrane	0.018458697
CC	GO:0098802	plasma membrane signaling receptor complex	0.018458697
MF	GO:0140375	immune receptor activity	0.00018479
MF	GO:0004896	cytokine receptor activity	0.000803343
MF	GO:0070851	growth factor receptor binding	0.002342202
MF	GO:0001540	amyloid-beta binding	0.00568427
MF	GO:0003953	NAD + nucleosidase activity	0.00568427
MF	GO:0050135	NAD(P) + nucleosidase activity	0.00568427
MF	GO:0061809	NAD + nucleotidase, cyclic ADP-ribose generating	0.00568427
MF	GO:0070412	R-SMAD binding	0.006992841
MF	GO:0016493	C-C chemokine receptor activity	0.006992841
MF	GO:0038187	pattern recognition receptor activity	0.006992841

**Table 4:** KEGG enrichment analysis

ID	Description	p.adjust
hsa04620	Toll-like receptor signaling pathway	3.02E-07
hsa04659	Th17 cell differentiation	7.07E-06
hsa04668	TNF signaling pathway	7.52E-06
hsa05235	PD-L1 expression and PD-1 checkpoint pathway in cancer	3.92E-05
hsa04657	IL-17 signaling pathway	4.85E-05
hsa04933	AGE-RAGE signaling pathway in diabetic complications	6.29E-05
hsa04625	C-type lectin receptor signaling pathway	7.30E-05
hsa04613	Neutrophil extracellular trap formation	9.15E-05
hsa04060	Cytokine-cytokine receptor interaction	9.54E-05
hsa04380	Osteoclast differentiation	0.000165879

### Construction of intersecting gene interaction networks

**Fig. 5:**
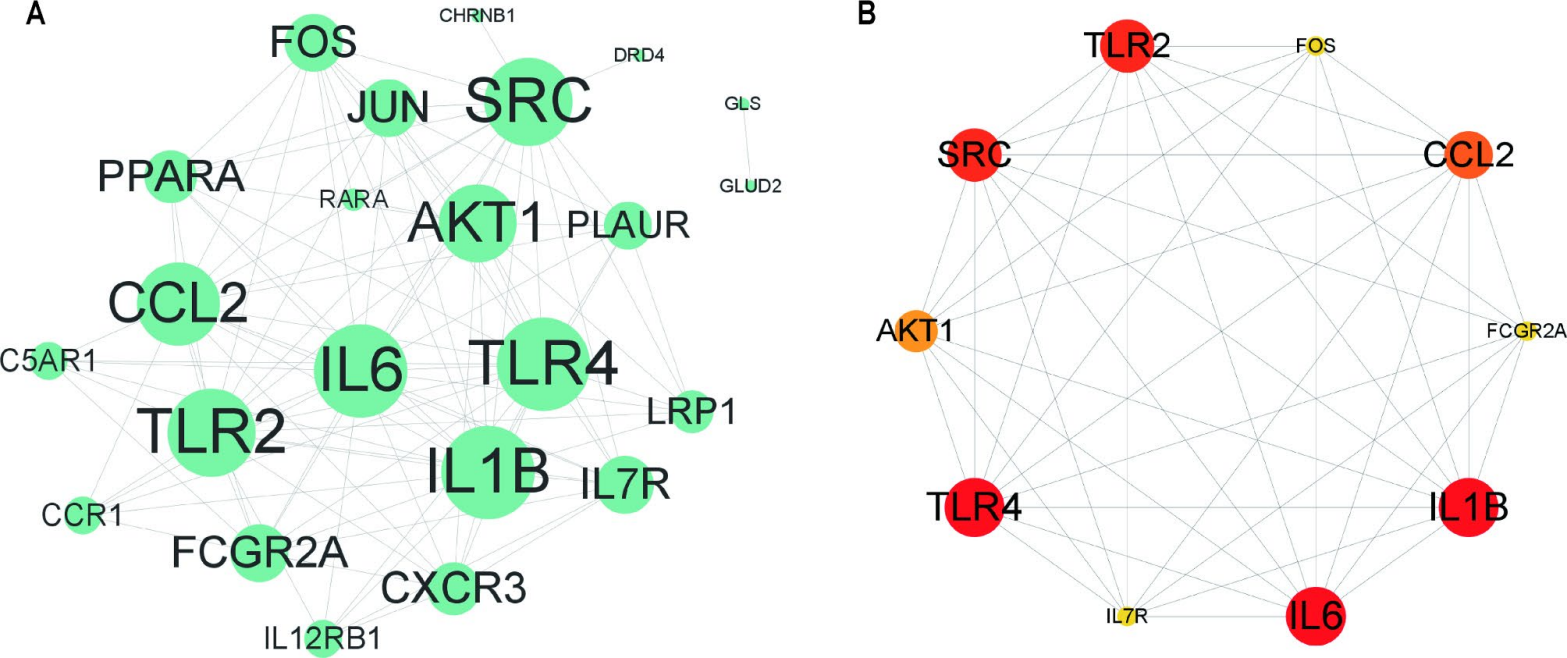
Intersecting gene interaction networks. (**A**) Graph of protein-protein interaction networks constructed from intersecting genes; node size indicates connectivity of nodes in network, larger nodes denote greater connectivity. (**B**) Graph of top 10 nodes in connectivity ranking; darker, larger nodes denote higher connectivity

To analyze the interactions between intersecting genes, we used the STRING database for protein interaction analysis of intersecting genes and constructed a protein-protein interaction (PPI) network related to intersecting genes, containing a total of 25 nodes and 105 edges, including the two isolated nodes CD320 and EDNRB. The results from the STRING database were imported into the Cytoscape software and the obtained data were evaluated for presentation, where the node size was proportional to the connectivity of the nodes (Fig. 5A). The cytoHubba plug-in was then used to analyze the connectivity to obtain the top 10 hub genes, with darker colors indicating greater connectivity of the node; the more nodes that were connected to the node, the more important it was in the network (Fig. 5B). The top six genes with the most interactions with the intersecting genes were IL6 (interactions with 17 intersecting genes), TLR4 (interactions with 17 intersecting genes), IL1B (interactions with 17 intersecting genes), SRC (interactions with 16 intersecting genes), TLR2 (interactions with 16 intersecting genes), and CCL2 (interactions with 15 intersecting genes).

### Immunological infiltration analysis

**Fig. 6:**
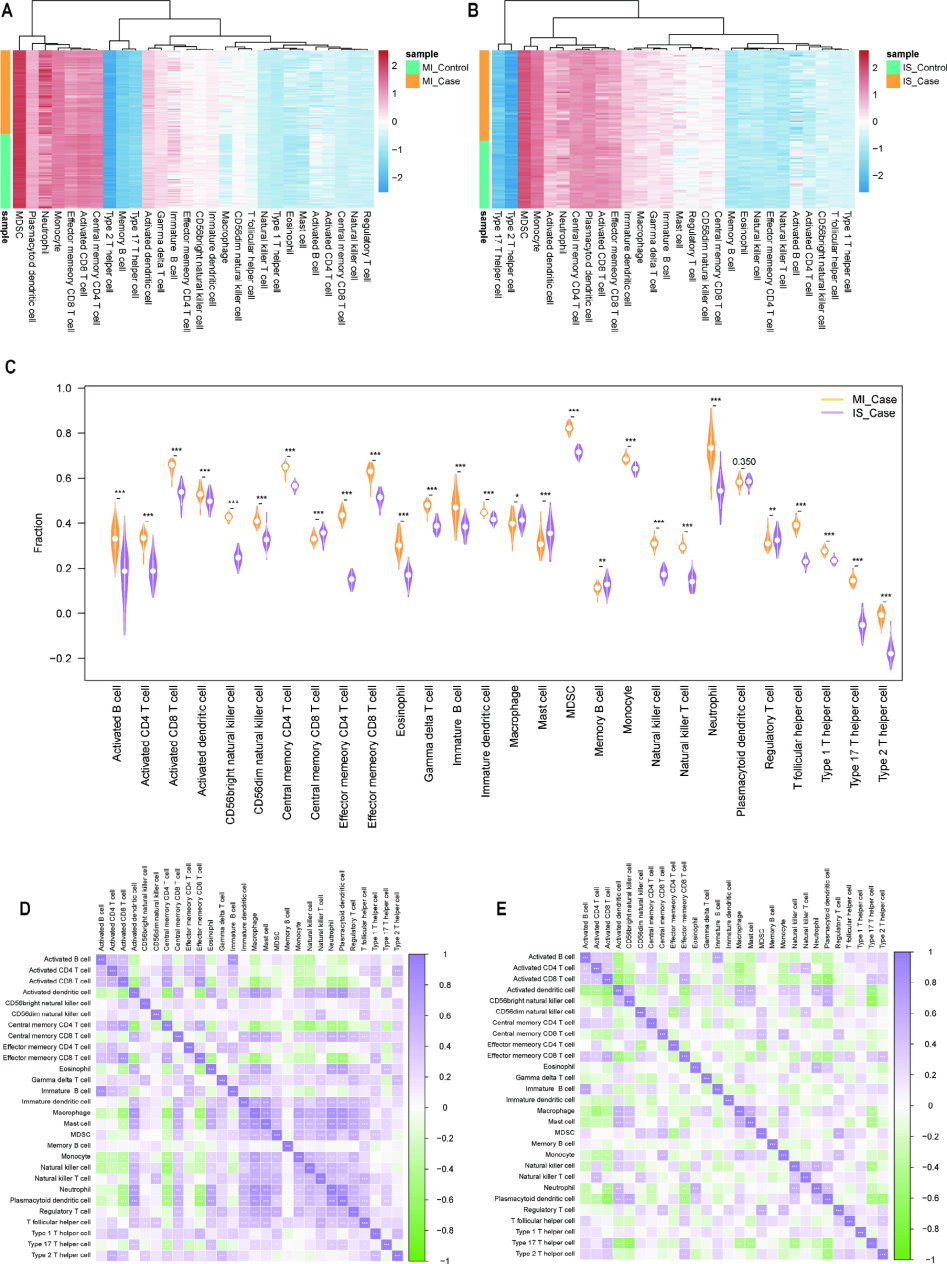
Immunocyte infiltration analysis. (**A**) Heat map of 28 types of immune cell infiltration in (**A**) MI and (**B**) IS datasets; x-axes represent 28 types of immune cells and warmer colors represent higher degree of immune infiltration. **C**: Box plot of immune cell expression differences between MI and IS; x-axis indicates 28 immune cell types and y-axis indicates degree of immune cell infiltration. Heat maps of immune cell correlation in (**D**) MI and (**E**) IS; x- and y-axes indicate 28 immune cell types and color indicates correlation; purple is positive correlation, green is negative correlation, and asterisks indicate correlation p-values. *p < 0.05; **p < 0.01; ***p < 0.001. IS: ischemic stroke; MI: myocardial ischemia

To analyze the role of immune genes in the MI versus IS data, we used single-sample gene set enrichment analysis (ssGSEA) to quantify the relative abundance of each immune cell infiltrate by labeling each infiltrating immune cell type. The infiltration scores of immune cells in the MI and IS data are displayed using a heat map to examine the expression of immune cells (Fig. 6A and B). The results showed that immune cells such as MDSC, monocytes, central memory CD4 + T cells, and plasmacytoid dendritic cells (pDCs) were highly expressed in the MI and IS data. Immune cells such as memory B cells, Th17 and Th2, mast cells, and macrophages were weakly expressed in the MI data and highly expressed in the IS data.

We used the R package vioplot to create violin plots to show the distribution of the fraction of each immune cell in the two diseases, and used Wilcox rank-sum test algorithm to calculate the difference in the degree of infiltration of the same immune cells in the two disease samples. Over 90% of the immune cells were differentially expressed in the MI and IS data, that is, with the exception of pDCs, the remaining immune cells were significantly different between the two diseases (Fig. 6C).

To investigate the relationship between immune cells in MI and IS, we analyzed immune cell correlations and plotted correlation heat maps using the R package corrplot to determine whether the correlation trends were consistent among immune cells in different diseases (Fig. 6D and E). In MI, pDCs were positively correlated with neutrophils (COR = 0.840), macrophages were positively correlated with neutrophils (COR = 0.816), and central memory CD4 + T cells were highly correlated with activated DCs (COR=-0.570). In the IS, immature B cells were highly positively correlated with activated B cells (COR = 0.642), effector memory CD8 + T cells were positively correlated with activated CD8 + T cells (COR = 0.612), and CD56 bright natural killer cells were highly negatively correlated with Th17 cells (COR = -0.631).

### Construction of TF-intersegmental gene networks and pathway intersegmental/immune gene networks

**Fig. 7:**
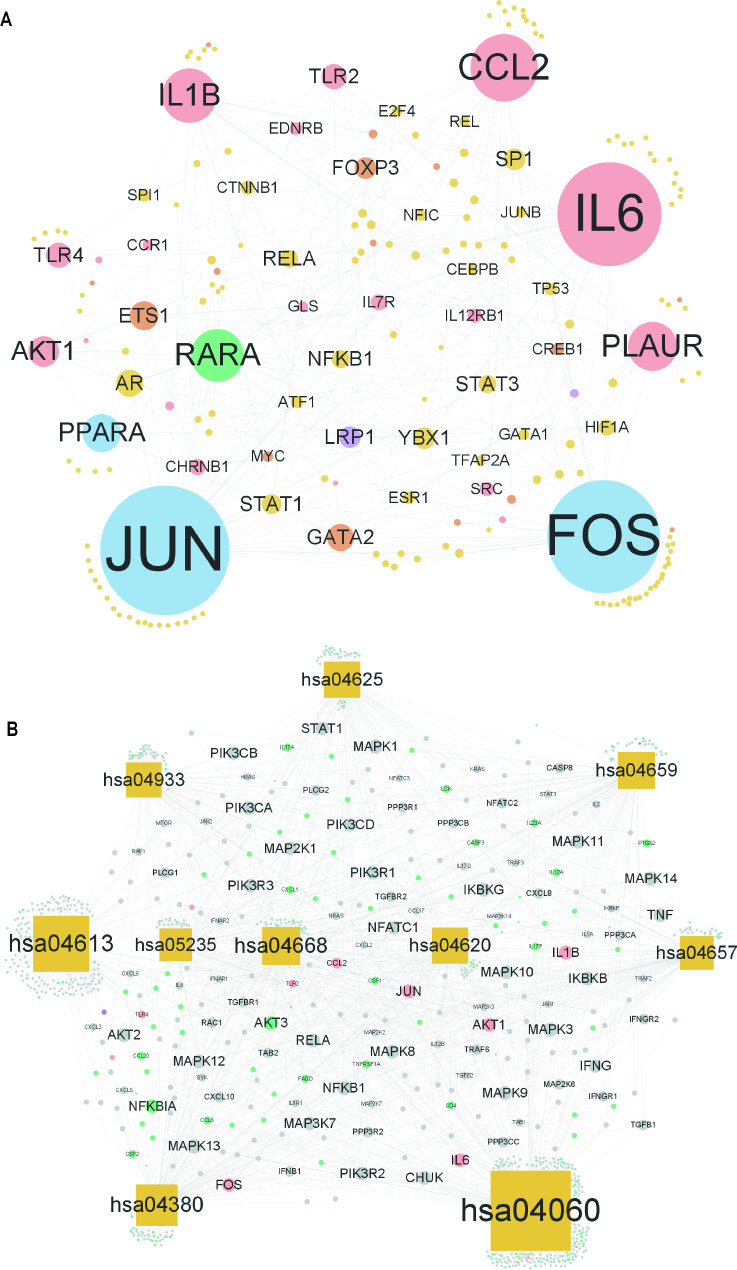
TF-intersecting gene network and pathway-intersecting gene/immune gene network. (**A**) TF-intersecting gene network; pink circles are intersecting genes, purple circles are intersecting genes-immune genes, blue circles are intersecting genes-TF, green circles are intersecting genes-immune genes-TF, yellow circles are TF, orange circles are TF-immune genes, and node size is positively correlated with node connectivity. (**B**) Pathway intersection gene/immune gene network; pink circles are intersection genes, purple circles are intersection genes-immune genes, green circles are immune genes, yellow squares are KEGG pathway, grey circles are other genes in the pathway, and node size is positively correlated with node connectivity. TF: transcription factor

To investigate the regulatory relationship between intersecting genes and TF, we downloaded the relationship between TF and target genes from a TF-related database and extracted the TF corresponding to intersecting genes. A TF-intersecting gene network was established using Cytoscape software, and the topological properties of the TF target network were analyzed (Fig. 7A). The network comprised 192 nodes and 397 edges. We also mapped 782 immune genes to the TF-intersection gene network and identified significant nodes based on topological analysis to illustrate the relationships between TFs, intersection genes, and immune genes. From the network, we found that genes such as JUN, FOS, and PPARA are intersection genes and TFs that regulate other genes to influence biological functions. LRP1 is an immune gene and TF, and RARA is an intersection and immune gene, as well as a TF that influences immune functions by regulating other genes.

To analyze the association of intersecting genes in important pathways, we extracted the top 10 KEGG pathways important for enrichment analysis and obtained all genes in these pathways using the R package clusterProfiler. These gene sets may contain a combination of intersecting, immune, or other genes in the pathway. We built a pathway gene network based on these gene properties using Cytoscape software (Fig. 7B) and used the pathways as a bridge to discover relationships between the intersecting and immune genes. FCGR2A is the intersection gene of MI and IS as well as the immune gene, regulating the osteoclast differentiation and neutrophil extracellular trap formation pathways. Genes in the pathway, intersecting genes, and immune genes influence the occurrence of MI and IS, providing the potential for crosstalk.

### Model construction and validation

**Fig. 8:**
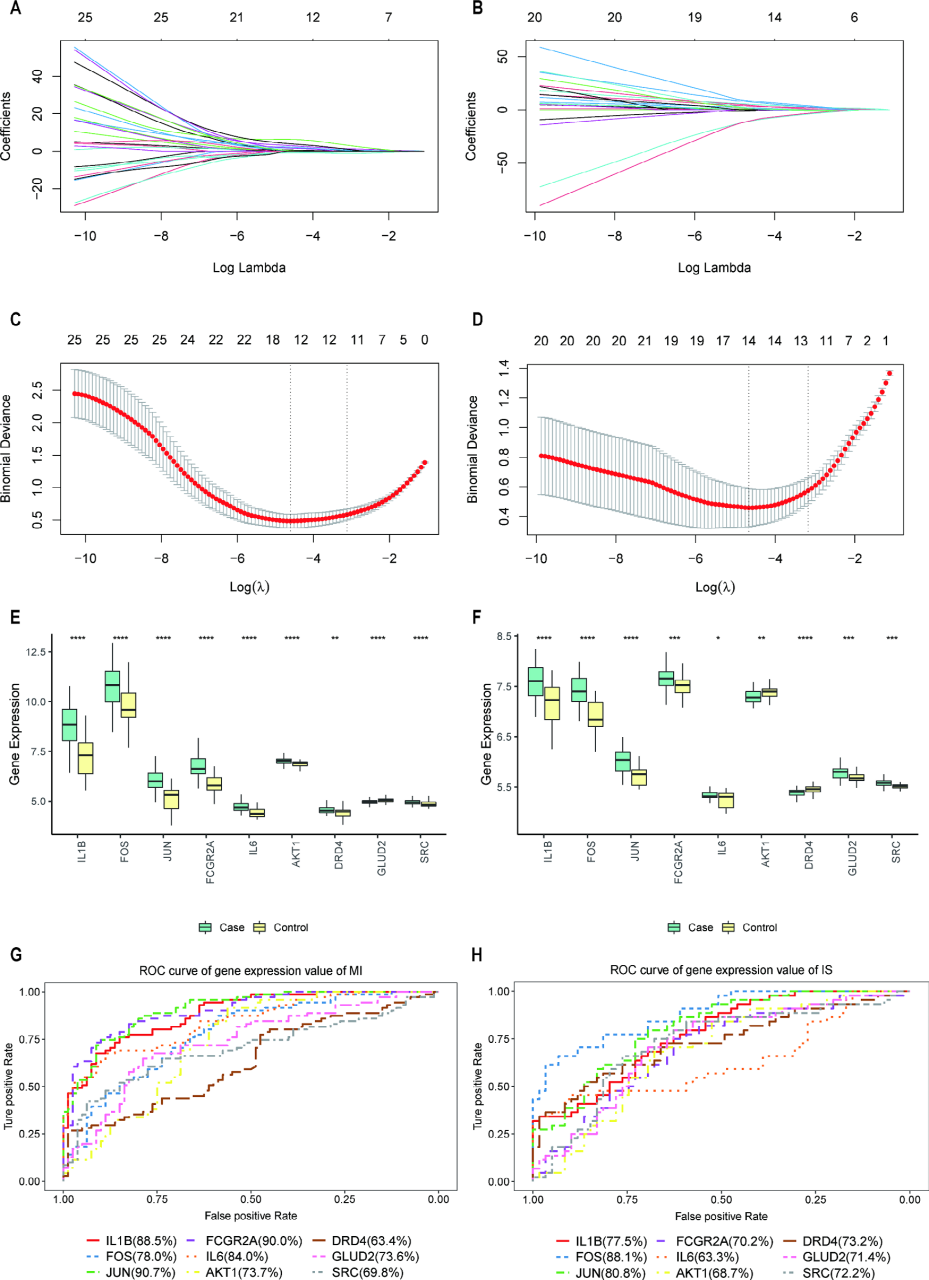
Model construction and validation. (**A, B**) MI and IS characteristic gene change curves; y-axis (above) is remaining number of variable genes whose variable coefficient is not zero at current logarithm of lambda. (**C, D**) MI and IS cross-check lambda results; there are two dashed lines in plot: one is minimum mean squared error and other is standard error of minimum mean squared error. (**E, F**) Box plot of differences in gene expression between MI and IS for IL1B, FOS, JUN, FCGR2A, IL6, AKT1, DRD4, GLUD2, and SRC; *p < 0.05; **p < 0.01; ***p < 0.001 (**G, H**) ROC curves for IL1B, FOS, JUN, FCGR2A, IL6, AKT1, DRD4, GLUD2, and SRC genes in MI and IS. IS: ischemic stroke; MI: myocardial ischemia

To better investigate the relationship between MI and IS and to predict the two diseases, we further screened the 25 intersecting genes. Based on the R package glmnet, intersecting genes were further screened using LASSO logistic regression for MI expression profile data and IS expression profile data (Fig. 8A, B, C, and D). Thirteen intersecting genes in MI and 14 in IS were obtained by screening, and they shared nine intersecting genes, IL1B, FOS, JUN, FCGR2A, IL6, AKT1, DRD4, GLUD2, and SRC, which were considered as potential biomarkers. The expression of these nine genes was significantly different between the disease and normal samples for both diseases, and most of the genes showed the same expression trend, with high expression levels in MI and IS disease samples (Fig. 8E and F), as plotted using the ggplot function for the differences in potential biomarkers in MI and IS. To investigate the predictive efficacy of these nine genes for both diseases, we performed receiver operating characteristic (ROC) analysis (Fig. 8G and H) and found that the area under the curve (AUC) of the remaining seven genes was > 65% for both MI and IS, except for IL6 (MI: 84.0%; IS: 63.3%) and DRD4 (MI: 63.4%; IS: 73.2%), indicating that the expression values of these genes for the prediction of MI and IS were reliable.

### Blood samples to verify the expression of hub genes

**Fig. 9:**
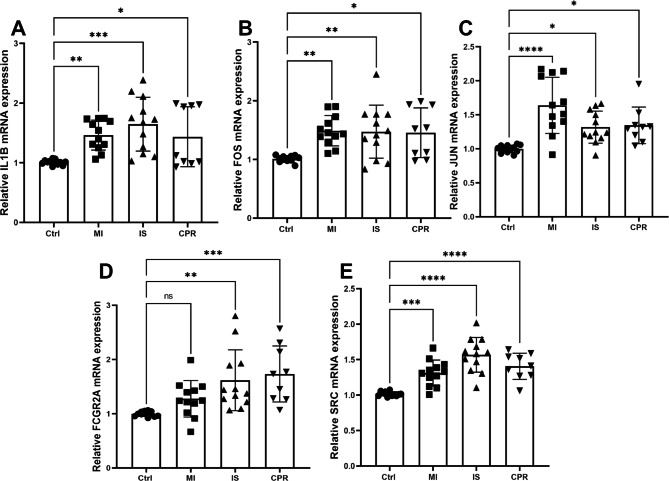
Blood samples to verify expression of hub genes. mRNA expression levels of (**A**) IL1B, (**B**) FOS, (**C**) JUN, (**D**) FCGR2A, and (**E**) SRC in blood specimens from patients with acute IS, acute MI, CPR, and normal physical examination. ^ns^p>0.05; *p < 0.05; **p < 0.01; ***p < 0.001; ****p < 0.0001. Ctrl: Control; IS: ischemic stroke; MI: myocardial ischemia; CPR: cardiopulmonary resuscitation

A total of 45 blood samples were collected, including 12 patients with acute IS, 12 with acute MI, nine with cardiopulmonary resuscitation, and 12 with normal physical examination; RT-qPCR results showed that the expression levels of IL1B, FOS, JUN, and SRC were significantly elevated in all groups of patients compared with normal physical examination (Fig. 9A, B, C, and E). FCGR2A expression levels were significantly elevated in patients with acute IS and cardiopulmonary resuscitation compared to that in normal subjects, while no significant abnormalities were observed in acute MI (Fig. 9D).

### Cellular model to verify the expression of hub genes

**Fig. 10:**
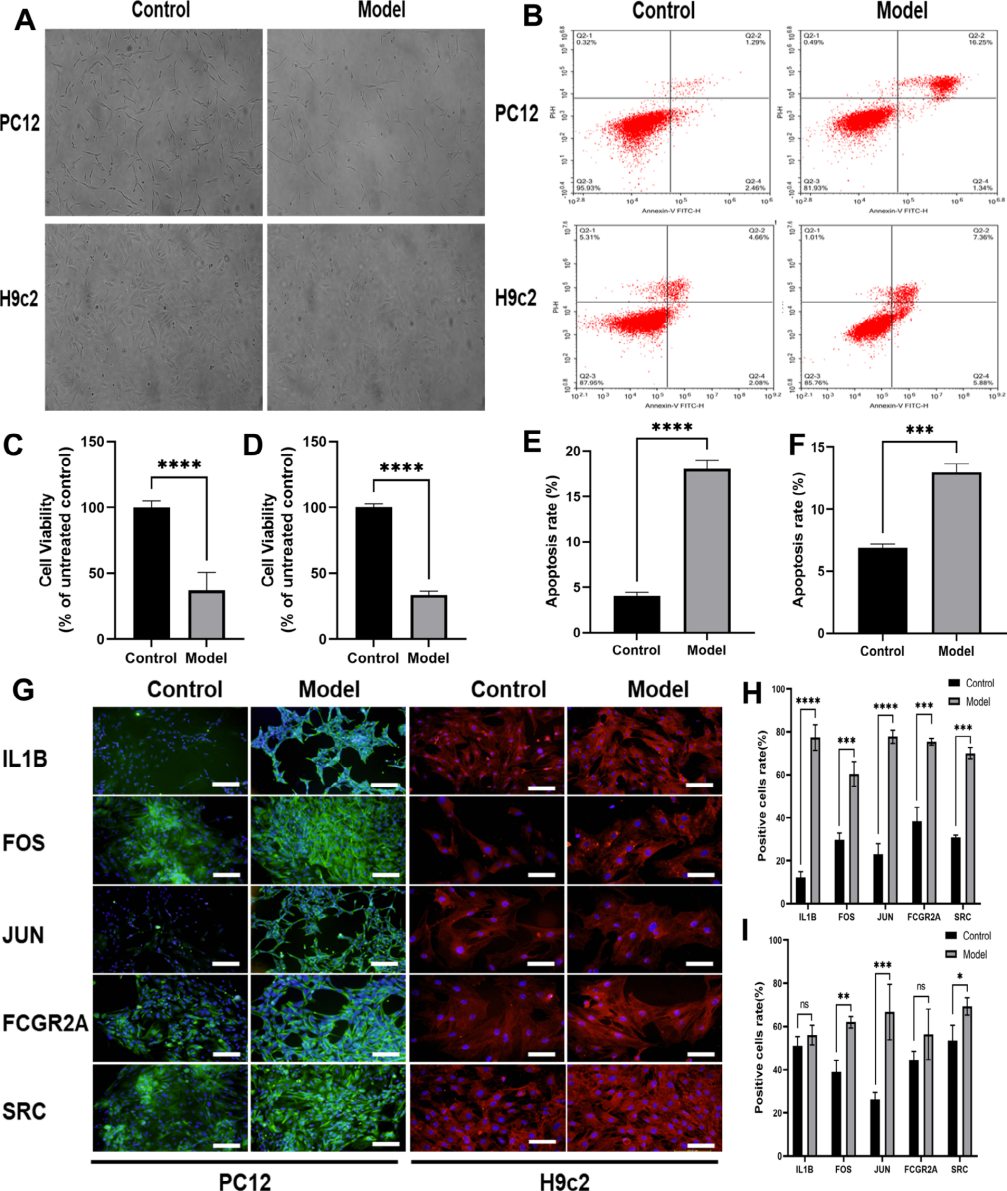
Cellular models to verify expression of hub genes. (**A**) Morphological changes of PC12 and H9c2 cells before and after cell modeling. PC12 cells had reduced cell morphology and longer tentacles compared with normal cells after modeling. H9c2 cells had reduced cell morphology and fullness compared with normal cells after modeling. (**B**) Flow cytometry detection of apoptosis of PC12 and H9c2 cells before and after cell modeling. (**C, D**) CCK-8 assay of cell viability before and after modeling of PC12 and H9c2 cells showed a decrease in cell viability after modeling. (**E, F**) Significant apoptosis was observed after modeling of PC12 and H9c2 cells. (**G**) Immunofluorescence staining detected expression of key genes in PC12 and H9c2 cells and hypoxia-reoxygenated cell models (bar = 50 μm). (**H, I**) Expression levels of IL1B, FOS, JUN, FCGR2A, and SRC genes were significantly higher in PC12 cells after modeling compared with controls, and differences were statistically significant. Expression levels of FOS, IL1B, FCGR2A, and SRC were significantly elevated after modeling of H9c2 cells, and the difference was statistically significant, whereas difference in JUN expression was not. Nuclei were stained blue with DAPI. ^ns^p>0.05; *p < 0.05; **p < 0.01; ***p < 0.001; ****p < 0.0001

We successfully established hypoxic reoxygenation models of PC12 and H9c2 cells using CCK-8 and flow cytometric assays for cell viability and apoptosis (Fig. 10A). CCK-8 results suggested that cell viability decreased after in both models (Fig. 10C and D). Flow cytometry indicated increased apoptosis (Fig. 10B, E, and F). Immunofluorescence staining was used to detect the expression of hub genes IL1B, FOS, JUN, FCGR2A, and SRC; their expression levels were significantly higher in PC12 cells after modeling compared with the controls, and the differences were statistically significant. The expression levels of FOS, IL1B, FCGR2A, and SRC were significantly elevated after modeling H9c2 cells, and the difference was statistically significant, whereas the difference in JUN expression was not significant (Fig. 10G, H, and I); negative control images are presented in Supplementary Material (Additional file 1: Figure S1).

## Discussion

Cardiocerebrovascular disease is a serious threat to human health. Despite extensive efforts to improve treatment, its protective effect on the heart and brain, as well as its prognosis, has not fundamentally improved [1]. The heart and brain, as terminally differentiated organs, are highly sensitive to injury and are difficult to repair after injury. The proportion of patients with comorbid cardiovascular disease is estimated to be > 70%, and a growing number of studies have confirmed that most molecular events that occur after neurological injury also occur after myocardial injury, suggesting a common mechanism of injury between the heart and brain [6, 16]. Therefore, it is crucial to explore the common injury signaling pathways in the heart and brain and to establish timely and early means and strategies for the protection of the heart and brain in the early stages of cardiac and cerebral injury. Cardiocerebral injury can occur in various ways; however, ischemia-hypoxia-reperfusion is the most important initiating factor leading to cardio-cerebral injury [17]. Many studies have confirmed that an important mechanism of ischemic-reperfusion injury is the excitatory neurotoxicity of GLU, which is further refined at the cellular level. The molecular events that occur after injury are complex and include oxidative stress, mitochondrial dysfunction, altered cell permeability, calcium overload, impaired energy metabolism, activation of apoptotic signals, and the release of inflammatory factors involved in the process of cell death after injury [18, 19]. GLU is a nonessential amino acid that maintains neuronal survival and synaptic plasticity under physiological conditions. However, under pathogenic conditions, the massive release of extracellular GLU can stimulate GLuR, induce inward calcium flow, and activate calcium-dependent death signaling pathways. In turn, GLU toxicity in cells and tissues is not immediately alleviated after resuscitation and may further exacerbate the damage [20–23]. Therefore, the two are closely related and may share a common mechanism of injury based on the mutual hemodynamic and pathophysiological mechanisms between the heart and brain. Combined with the currently identified role of GLuR in the process of cardiac and cerebral ischemia-hypoxia-reperfusion, the present study explored the relevant roles and mechanisms of GLuR-related crossover genes in the process of cardiac and cerebral ischemia and explores the role of GLuR-related crossover genes in the process of cardiac and cerebral ischemia through LASSO model construction. The current study investigated the role and mechanism of GLuR-related crossover genes in MI and IS and explored the common crossover genes in MI and IS using LASSO model construction, blood samples, and cellular models to verify the expression of hub genes and predict the common regulatory pathways of both diseases.

In the present study, we obtained four datasets, GSE66360, GSE48060, GSE16561, and GSE22255, applied bioinformatic methods to first analyze the DEGs for MI and IS, and then took intersections with GLuR genes to obtain 25 intersecting genes. Interplay network construction, immune infiltration analysis, intersection gene-TF, and pathway intersection gene/immune gene interplay network construction were also performed on the intersection genes, and the roles and mechanisms of the GLuR-related intersection genes in MI and IS were explored. Finally, through LASSO model construction, nine genes, including IL1B, FOS, JUN, FCGR2A, IL6, AKT1, DRD4, GLUD2, and SRC, were identified as potential biomarkers. Furthermore, the differential box line plots of potential biomarkers in MI and IS were plotted by the ggplot function, and it was found that except for AKT1, DRD4, and GLUD2, the expression of the remaining six genes were significantly different between disease samples and normal samples. To investigate the predictive efficacy of these nine genes for the two diseases, we performed ROC analysis and found that except for IL6 (MI: 84.0%; IS: 63.3%) and DRD4 (MI: 63.4%; IS: 73.2%), the AUC of the remaining seven genes was > 65% in both MI and IS. It was further confirmed by clinical blood samples and cellular models that the expression of relevant hub genes was consistent with the bioinformatics analysis. Taken together, these results suggest that IL1B, FOS, JUN, FCGR2A, and SRC can be potential biomarkers for combined cardiocerebral injury, laying the foundation for the discovery of new targets and therapeutic options.

IL1B is a member of the IL1 cytokine family, which is produced by activated macrophages as a pre-protein, and is hydrolyzed by cystathionine aspartase 1 into its active form. This cytokine is an important mediator of the inflammatory response and is involved in a variety of cellular activities including cell proliferation, differentiation, and apoptosis through the NF-κB and TLR signaling pathways [24, 25], which induces prostaglandin synthesis, neutrophil inflow and activation, T cell activation, cytokine production, B cell activation, antibody production, fibroblast proliferation, collagen production, and Th17 cell differentiation [26, 27]. IL1B is an important marker of inflammation, and disruption of IL1B homeostasis plays a crucial role in the development of atherosclerosis [28]. Previous studies have shown that ischemia/reperfusion injury to the central nervous system can lead to the activation of inflammatory factors, which induces the release of pro-inflammatory factors and chemokines that exacerbate the inflammatory response and ultimately lead to neuronal death [29]. A biochemical analysis of acute myocardial infarction identified IL1B as a biomarker of acute myocardial infarction that mediates the inflammatory response after acute myocardial infarction [30–32]. These studies are consistent with the present study, indicating that IL1B can participate in the biological process after ischemia-reperfusion injury in cardiovascular and cerebrovascular diseases, thereby changing the endpoint of cardiovascular and cerebrovascular disease events.

The FOSgene, also known as the AP-1 transcription factor subunit, encodes a leucine zip protein that dimerizes with proteins of the JUN family to form the transcription factor complex AP-1. FOS is thought to prevent the inflammatory response and neuronal death that occur during IS and brain injury and is a neuroprotective factor [33, 34], and c-FOS is activated during myocardial remodeling after MI-reperfusion injury, thereby prolonging apoptosis in cardiomyocytes, and is considered to be an anti-apoptotic factor [35, 36], suggesting that FOS plays an equal role in MI and IS.

The JUN gene, also an AP-1 transcription factor subunit, encodes a protein that is highly similar to viral proteins and interacts directly with specific target DNA sequences to regulate gene expression. It is involved in cell growth, development, and differentiation under normal conditions but is expressed at low levels. In the early stages of MI, researchers have found elevated expression levels of c-FOS and c-JUN, referred to as immediate early genes [37], endoplasmic reticulum stress and soluble epoxide hydrolase activation after MI-reperfusion injury are closely associated with the phosphorylation levels of JUN [38]. Similarly, in animal models of cerebral ischemia-reperfusion injury, a previous study found that JUN can be a marker for the progression of cellular injury, and after IS, the number of significantly expressed JUN-positive cells increased, and hypothermia can slightly reduce cerebral infarction after ischemia for reasons related to JUN expression [39, 40].

The FCGR2A (Fc fragment of the immunoglobulin G receptor IIa) gene encodes a member of the immunoglobulin FC receptor gene family found on the surface of many immune-responsive cells. The protein is a cell surface receptor found on phagocytes such as macrophages and neutrophils, which are involved in the phagocytosis and clearance of immune complexes. FCGR2A is an inflammation-related gene. Similar to the association of the rs1137101 gene polymorphism with myocardial infarction [41], the gene polymorphism of FCGR2A has also been confirmed to be associated with susceptibility to IS in people of different races [42, 43]. C-reactive protein (CRP) plays a critical role in atherosclerosis, and FCGR2A is its main receptor. When bound to CRP, FCGR2A induces monocyte-endothelial cell interactions, upregulates the expression of monocyte chemotactic protein 1 and endothelin-1 in endothelial cells, induces the release of cytokines from monocytes, stimulates the release of matrix metalloproteinases, and increases the phagocytosis of oxidized low-density lipoproteins by macrophages [44]. Calverley et al. [45] found increased expression levels of platelet surface FCGR2A in patients with unstable angina, acute myocardial infarction, IS, and in high-risk individuals carrying two or more atherosclerotic risk factors. The expression of FCGR2A on platelet surfaces is also increased in individuals with two or more risk factors for atherosclerosis and is more pronounced in patients with diabetes. In the present study, we found that FCGR2A is a crossover gene between MI and IS and an immune gene that regulates osteoclast differentiation and neutrophil extracellular trap formation pathways.

SRC is a non-receptor tyrosine kinase that can be activated in vivo by cytokine receptors, tyrosine kinase receptors, G protein-coupled receptors, integrin receptors, and a variety of cellular emergency signals to participate in cell adhesion migration, proliferation and differentiation, angiogenesis, and intracellular transport [46, 47]. In brain injury, SRC kinases participate in the destruction of the vascular barrier, formation of cerebral edema, and neovascularization after IS through various signaling pathways and target genes. For example, SRC expression levels are increased after IS, and SRC kinases can mediate the over-activation of NMDA receptors [48]. Numerous studies have shown that SRC is involved in signal transduction during major pathophysiological processes in the heart and that SRC activity is closely related to the maintenance of cardiovascular homeostasis. The phosphorylation status of SRC is altered in response to various injurious factors or stress stimuli and regulates different cardiac pathological states, such as hypertension, coronary heart disease, ischemic heart disease, MI-reperfusion injury, arrhythmias, and cardiomyopathy, by modulating cell growth, differentiation, motility, function, and electrophysiological signaling. Hypoxia/reoxygenation promotes the autophosphorylation of SRCs associated with the surface of neonatal rat cardiac myocytes at an early stage [49], and the JNK/SAB/SRC/ROS pathway reduces mitochondrial-phosphorylated SRC expression and increases mitochondrial ROS levels [50]. Moreover, SRC activation plays an important protective role against MI-reperfusion injury and is a potential target for its treatment [51].

GO functional enrichment analysis showed that the intermingled genes were mainly enriched in the outer plasma membrane, secretory granule membrane, the cellular fraction of the plasma membrane signaling receptor complex, through immune receptor activity, cytokine receptor activity, growth factor receptor binding, amyloid-β binding, NAD + nuclease activity, NAD(P) + nuclease activity, other molecular functions mainly involved in responses to lipopolysaccharide and bacterial-derived molecules, positive regulation of lymphocyte and leukocyte activation, intercellular adhesion, and cell activation, among other biological processes. KEGG analysis showed that the intersecting genes were mainly involved in the TLR signaling pathway, Th17 cell differentiation, and TNF, which are a class of pattern recognition proteins that play an integral role in the regulation of systemic inflammatory responses and play a key role in activating the inflammatory cascade after hypoxic-ischemic events, and subsequently contribute to the neuroprotective or deleterious effects of CVD-induced neuroinflammation. They also play key roles in the development and progression of atherosclerosis. After the onset of IS and MI, TLR signaling pathways and downstream cascades trigger immune responses through the production and release of various inflammatory mediators and are expected to be targeted for the treatment of cardiovascular and cerebrovascular diseases [52, 53], which is consistent with our findings. Th17 cells differentiate from CD4 + T cells in response to TGF-β and IL6 and can produce proinflammatory cytokines. However, unstable plastic Th17 cells can also transdifferentiate into Tregs, thereby reducing inflammation, and in myocardial infarction disease, myocardial infarction-associated transcripts can promote cellular differentiation of Th17 by upregulating Th17-related genes, again suggesting a role for Th17 cell differentiation in MI [54]. Thus, Th17 cells play a dual role in the post-ischemic inflammatory response. Previous studies have shown that Th17 cells are closely associated with cognitive impairment, stroke recurrence, and mortality in acute stroke patients [55]. In acute myocarditis, Th17 cells characterized by the production of IL17 were similarly found to be a feature of the acute phase [56], but whether they can be involved in regulating the cardio-cerebral co-protection process needs to be further explored. In the present study, we also reported other related enrichment pathways that provided ideas for our subsequent study on cardio-cerebral co-protection.

The results of the PPI network associated with intersection genes suggested that the top six genes with the most interactions with intersection genes were IL6 (interactions with 17 intersection genes), TLR4 (interactions with 17 intersection genes), IL1B (interactions with 17 intersection genes), SRC (interactions with 16 intersection genes), TLR2 (interactions with 16 intersection genes), and CCL2 (these results are consistent with our model construction and functional enrichment results, which can be used as a reference for further studies).

The results of the immune infiltration analysis suggested that 90% of the immune cells were differentially expressed in the MI and IS data, and immune cells such as MDSC, monocytes, central memory CD4 T cells, and pDCs were highly expressed in the MI and IS data. Memory B cells and MDSC, including immature granulocytes, macrophages, and DCs at different stages of differentiation, are well-known immunosuppressive cells that play a decisive role in many disease states [57]. Recent studies have reported that MDSC can be recruited to the infarcted myocardium during the acute phase of acute myocardial infraction to induce the secretion of protein hydrolases and promote apoptosis [58–60]. In a mouse IS model, IS was found to increase the number of PMN-MDSC-LCs in the bone marrow, spleen, and ischemic hemisphere, further suggesting an important role of MDSC in cardiac and cerebral ischemia [61]. The absence of CD4 + T cells leads to an increase in monocyte count in infarcted myocardial cells, suggesting that CD4 + T cell infiltration into the myocardial infarct site may induce pro-inflammatory monocyte differentiation [62]. Regulatory T cells (Tregs) have an important role in cardiac tissue repair after myocardial infarction, thereby regulating the post-infarction inflammatory response and severe ventricular remodeling, protecting cardiomyocytes from apoptosis, and promoting myocardial healing [63, 64]. These findings suggest that the infiltration of different immune cell types is closely related to the progression of cerebral ischemia and myocardial ischemic diseases. It is worthwhile to further explore the immune mechanisms associated with the immune infiltration results found in the current study.

This study had some limitations. To obtain more accurate conclusions and bioinformatics analysis verification, more datasets are required. We are currently conducting relevant sample collection and sequencing work. Furthermore, we only verified the expression of the hub genes in blood samples and cell models and did not further confirm their mechanisms of action. The conclusions drawn here should be verified in subsequent experiments. Since the nature of the study design was retrospective, some important clinical information could not be obtained and analyzed in combination with clinical information, such as the relationship between related genes and onset time, and the relationship with prognosis. Further analysis combined with clinical information is one of the purposes of our study, which can provide a direction and basis for future clinical studies.

## Conclusions

In the current study, we found that the GLuR-related genes IL1B, FOS, JUN, FCGR2A, and SRC were expressed in MI and IS with the same trend, which can be used to predict the occurrence of cardiac and cerebral ischemic diseases and provide reliable biomarkers to further explore the co-protective mechanism after cardiac and cerebral ischemic injuries.

## Materials and methods

### Data acquisition and acquisition of GLuR-related genes

From the Gene Expression Omnibus (GEO https://www.ncbi.nlm.nih.gov/geo/) database [65] using the GEOquery package [66]. The MI-related datasets GSE66360 [67] and GSE48060[68] were downloaded. The GSE66360 dataset from Homo sapiens on GPL570 contained 99 samples 50 controls and 49 diseased), and the GSE48060 dataset from Homo sapiens on GPL570 contained 52 samples (21 controls, and 31 diseased).

The IS-related dataset GSE16561 was downloaded using R packages GEOquery [69]and GSE22255[70]. The GSE16561 dataset from *Homo sapiens* on the data platform GPL6883, contains 63 samples, including 24 control and 39 disease samples. Because the expression profile provided by GSE16561 is quantile-normalized data and contains negative values, the original expression matrix of GSE16561 (GSE16561_RAW.txt.gz) was downloaded from the GEO database, log-transformed, and normalized using the R package limma [71]. The GSE22255 dataset from *Homo sapiens* on the data platform GPL570 contains 40 samples (20 controls and 20 diseased). Because both MI and IS had two sets of data available, we combined them separately for subsequent analyses. To reduce variation across the combined samples, the R package sva was used [72], and the ComBat method was used to de-batch the combined data. After calibration, the MI data contained 80 disease and 71 control samples, whereas the IS data contained 59 disease and 44 control samples.

GLuR-related genes were collected from the GeneCards (https://www.genecards.org/) database [73], which provides comprehensive information on human genes by integrating gene-centric data from approximately 150 web-based sources, including genomic, transcriptomic, proteomic, genetic, clinical, and functional information. The term “glutamate receptor” was used as a search term to obtain a total of 5925 genes, and a screening score of > 20 yielded 239 genes as GLuR-related genes, as listed in Additional file 2: Table S1.

### DEG and gene enrichment analyses (GO and KEGG)

To analyze the effect of gene expression values on MI- and IS-related diseases, the two sets of data were analyzed separately for differences in grouping using the R package limma, based on the grouping information of cases and controls in the data. The genes were screened by setting an adj.p-value < 0.01 as the threshold, where genes with logFC > 0 and adj.p-value < 0.01 were DEGs with upregulated expression and genes with logFC < 0 and adj.p-value < 0.01 were DEGs with downregulated expression. The results of the DEG analysis were displayed as volcano plots using the R package ggplot2. The MI-DEGs, IS-DEGs, and GLuR were intersected, and the resulting intersected genes were crosstalk genes, which were displayed by the R package Venn plotting.

GO [74] annotation analysis and KEGG [75] pathway enrichment analysis was performed on intersecting genes by using the R package cluster Profiler [76], with a threshold value of < 0.05 for FDR considered statistically significant, entry screening criteria of adj.p-value < 0.05 and q-value < 0.05, and p-value correction by the Benjamini-Hochberg method (BH).

### Intersectional gene interaction network construction

The STRING database (https://string-db.org/) [77] was used to construct a PPI network for the intersection of MI DEGs, IS DEGs, and GLuR-related genes, setting the parameter coefficient to 0.4. The PPI results were exported from the STRING database, the interactions were detected using Cytoscape [78] visualization, and the CytoHubba plugin was used for [79] analysis of hub genes in PPI networks.

### Immuno-infiltration analysis

Immune-related genes were downloaded from the literature PMID:28,052,254 [80], and the gene set contained 782 genes and 28 cell types, such as activated CD8 + T cells, activated DCs, macrophages, natural killer T cells, Tregs, and many other human immune cell subtypes. The R package GSVA [81] was used to analyze the degree of immune cell infiltration using (single-sample gene set enumeration analysis) algorithm for MI and IS expression profile data. Immune infiltration heat maps were plotted using the R package pheatmap, vioplot plotted differences in immune cell expression between the MI and IS disease groups, and corrplot plotted immune cell correlations.

### Intersectional gene-TF, pathway intersectional gene, and immune gene interaction network construction

TFs control gene expression by interacting with target genes post-transcriptionally. Transcriptional regulatory relationships unraveled by sentence-based text mining(TRRUST) (https://www.grnpedia.org/trrust/) are manually annotated databases of transcriptional regulatory networks [82]. The TRRUST database contains 800 human and 828 mouse TFs with 8444 human and 6552 mouse TFs target regulatory pairs. Open-access repository of transcriptional interactions (ORTI) http://orti.sydney.edu.au/about.html) is an integrated transcriptional interaction database, and its data are obtained from publicly available TF-TG interaction databases (i.e., HTRI, TFactS, TRED, TRRD, PAZAR, and NFI regulome) as well as mammalian TF and related TG retrieved from the literature [83]. Human transcriptional regulation interactions (HTRIdb) (http://www.lbbc.ibb.unesp.br/htri/) is a database containing experimentally validated TF-TG interactions in humans, including 284 TF, 18,302 genes, and 51,871 TF-TG regulatory relationships [84].

To analyze the regulatory role of TF in intersection genes, we downloaded TF-TG interactions from the TRRUST, ORTI, and HTRIdb databases and integrated the contents of the three databases to extract transcription factors targeting intersection genes and construct intersection gene-TF interaction networks. The intersection gene-TF interaction network was visualized using Cytoscape software.

To analyze the importance of intersecting genes in pathway enrichment, we obtained important KEGG pathways corresponding to intersecting genes through the functional enrichment analysis described above, extracted all genes under these pathways in the KEGG database using the R package clusterProfiler download, and tagged the genes types (intersecting, immune, and other pathway genes). The pathway intersection gene/immune gene network was constructed based on these gene properties and visualized using Cytoscape software.

### LASSO model construction

Minimizing the absolute shrinkage and selection operator LASSO regression is a machine learning algorithm commonly used to construct diagnostic models today, using regularization to address the occurrence of overfitting during curve fitting and to improve the accuracy of the model. To obtain the most relevant intersection genes, we used the glmnet package [85] to model MI and IS expression profile data, with intersection genes as independent variables and case/control as dependent variables. The model parameter is set to seed (3) and family = “binomial.” Intersecting genes in MI and IS were screened separately using LASSO regression, and shared intersecting genes were tagged as potential biomarkers. The expression values of potential biomarkers were extracted from all MI and IS samples, and difference box line plots were plotted using the ggplot2 package to analyze whether there were significant differences between the disease and control samples. ROC analysis of potential biomarkers was performed using the pROC package [86] to predict the disease prediction efficiency.

### Collection of human blood samples

A total of 45 relevant human blood samples were collected from March to December 2022, including 12 patients with acute IS, 12 with acute MI, 9 with cardiopulmonary resuscitation, and 12 with the normal physical examination; all blood specimens were collected according to the time of patient onset and were controlled within 24 h of onset, stored at 4 °C after collection, and processed within 24 h, using blood: red blood cell lysis solution (R1010, Solarbio China) (volume ratio) = 1:3, lysed on crushed ice for 30 min, centrifuged at room temperature, centrifuged at 3000 rpm for 10 min, discarded the red supernatant, added 1–2 mL TRIzol (CW0580S, CWBIO China), and frozen at -80 °C until.

### RT-qPCR validation

The mRNA levels of the selected genes were determined using RT-qPCR. A ultrapure RNA extraction kit (CW0581M; CWBIO, China) was used, according to the manufacturer’s instructions. cDNA was obtained using a HiScript II Q RT SuperMix for qPCR Reverse Transcription Kit (R223-01; Vazyme, China) according to the manufacturer’s protocol. RT-qPCR was performed using ChamQ Universal SYBR qPCR Master Mix (Q711-02, Vazyme China) according to the manufacturer’s protocol on a fluorescent PCR instrument (CFX Connect™ Real-Time, Burroughs Life Medical Products Co., Shanghai, China) Briefly, each reaction contained 5 µL of premix (2X), forward and reverse primers (1000 nM each), 10 ng of cDNA and an appropriate amount of nuclease-free water brought to a final volume of 10 µL. Three replicates were analyzed for each sample. Reactions were run with the following thermal cycling parameters: pre-denaturation 95 °C for 10 min for 10 s (denaturation), 60 °C for 30 s (annealing), and 72 °C for 30 s (extension). A final dissociation curve (melting curve) was then made and the PCR plates were kept at 4 °C until being removed from the machine. β-actin was used as the endogenous reference and the relative expression of the selected genes was calculated using the 2^−ΔΔCt^ method. The primers used in this study are listed in Table 5.

**Table 5:** Primer sequences

Primer	Primer sequences (5’-3’)	Product length(bp)	Annealing temperature(℃)
IL1B F	ATGATGGCTTATTACAGTGGCAA	132	60
IL1B R	GTCGGAGATTCGTAGCTGGA		
FOS F	GGGGCAAGGTGGAACAGTTAT	126	60
FOS R	CCGCTTGGAGTGTATCAGTCA		
JUN F	CGCAAACCTCAGCAACTT	248	60
JUN R	TCCGCTCCTGGGACTCCA		
FCGR2A F	TTTGAGATGAGTAATCCCAGCCA	112	60
FCGR2A F	TCAGGCCCCAGTCTCCATTTTA		
SRC F	TGGCAAGATCACCAGACGG	100	60
SRC R	GGCACCTTTCGTGGTCTCAC		
β-actin F	TGGCACCCAGCACAATGAA	186	60.8
β-actin R	CTAAGTCATAGTCCGCCTAGAAGCA		

### Establishment of cell culture and hypoxia-reoxygenation model

Rat neuronal PC12 cells (CL-0481; ProCell Life Science&Technology Co. Ltd., Wuhan, China) and rat cardiomyocytes H9c2 cells H9c2 (iCell-r012; iCell Bioscience Co. Ltd. Shanghai, China) were used, cultured in DMEM medium containing 10% fetal bovine serum, and passaged in an incubator at 37℃ and 5% CO2, and the medium was changed once at an interval of two days, and cells with 80% growth fusion were taken for passaging. Cells were divided into control and model groups with reference to previous methods [87].To establish a hypoxic reoxygenation model, cells were placed in sugar-free medium for 30 min, incubated in a hypoxic incubator (1% O2, 5% CO2, and 94% N2) for 6 h, and then the medium was replaced with a high-sugar medium containing 15% fetal bovine serum and 1% penicillin. Finally, cells were reoxygenated and reglycemic incubated for 6 h.

### CCK-8 for cell viability assay

Cells were seeded into 96-well cell culture plates with 100 µL of cell culture medium per well and treated according to the control and model groups. CCK-8 solution (10 µL) was added to each well and incubated at 37℃ for 2.5 h. The OD was determined at 450 nm. The cell survival rate of the control group was set to 100%, and changes in the cell survival rate of the remaining groups were analyzed.

### Flow cytometry

Flow cytometry was performed to detect apoptosis in each group. The cells were suspended in ice-cold PBS solution, and 1 × 10^6^ cells were collected from each group, centrifuged at 1000 rpm for 5 min, collected, mixed thoroughly with 300 µL of Binding buffer. Next, 5 uL of Annexin V-FITC and 10 uL of propidium iodide were added for 20 min under light-proof conditions. Finally, an additional 200 µL of Binding buffer was added and mixed thoroughly before performing flow cytometry (NovoCyte 2060R, Eisen Bio, Co., Ltd. Hangzhou, China).

### Immunofluorescence

Immunofluorescence was used to detect the positivity rate of selected molecules in each group of cells. The cells were fixed with 4% paraformaldehyde for 15 min, After the cell antigen was repaired and penetrated by Triton X-100 for 10 min, the cells were incubated with the following primary antibodies against: IL1B (AF7209; Beyotime China), FOS (ab222699; Abcam, Waltham, MA, USA), JUN (ab40766; Abcam), FCGR2A (HPA010776; Atlas Antibodies, Sweden), SRC (ab47505; Abcam), and IL6 (SAB5700632; Sigma-Aldrich, Germany) at 37 °C for 2 h. Primary antibody dilutions were set as follows: IL-1B, 1:100; FOS, 1:100; JUN,1:100; FCGR2A, 1:200; SRC, 1:100; IL6,1:200. PC12 cells were incubated with FITC-labeled secondary antibody (Invitrogen, Waltham, MA, USA) and H9c2 cells were incubated with Alexa-labeled fluorescent secondary antibody (Invitrogen) at 37 °C for 1 h. Secondary antibody dilutions were set as 1:200. Nuclei were stained with 0.5 µg/ml concentration of DAPI (Southern Biotech, Birmingham, Alabama, USA). The cells were imaged with a fluorescent microscope (Nikon ECLIPSE Ni-U/DS-Ri2, Nikon Co., Ltd. Kanagawa, Japan). The positive cell rate (five randomly selected high-power fields of view with at least 100 cells/group) was calculated as number of positive cells/total number of cells × 100.

### Statistical analysis

The bioinformatics analysis was based on R software (version 4.1.1, https://www.r-projec t.org/). Correlation analysis was performed using the Pearson’s method. Comparisons between two groups were performed using the Wilcoxon rank-sum test, whereas comparisons between three or more groups were performed using the Kruskal-Wallis test. ROC curves were plotted and the AUC was calculated using the pROC package. The results in Figs. 9 and 10 are presented as means ± standard deviation (SD). The datasets were tested for the normality of distribution using the Bonferroni test. Student’s t-test was used to compare normally distributed data groups (two groups). One-way analysis of variance (ANOVA) was used to compare groups. If not specifically indicated, p < 0.05 was considered significant.

## Electronic supplementary material

Below is the link to the electronic supplementary material.

Supplementary Material 1

Supplementary Material 2

## Data Availability

The datasets presented in this study can be found in online repositories. the names of the repository/repositories and accession number(s) can be found in the article/Supplementary Material.

## Abbreviations

MI: Myocardial ischemia
IS: Ischemic stroke
MDSC: Myeloid-derived suppressor cells
NMDAR: N-methyl-D-aspartate receptor
DEGs: Differentially expressed genes
KEGG: Kyoto Encyclopedia of Genes and Genomes
GEO: Gene Expression Omnibus
GLuR: Glutamate receptor-related genes
GO: Gene Ontology
BP: Biological process
MF: Molecular function
CC: Cellular component
BH: Benjamini-Hochberg method
PPI: protein-protein interaction
TME: The tumor microenvironment
SsGSEA: Single sample gene set enumeration analysis
TF: Transcription Factor
TRRUST: Transcriptional Regulatory Relationships Unraveled by Sentence-based Text mining
ORTI: Open-access Repository of Transcriptional Interactions
HTRIdb: Human transcriptional regulation interactions
LASSO: Least absolute shrinkage and selection operator
ROC: Receiver operating characteristic
AUC: Area under the curve
