# Supplementary material for: Bioinformatics and experimental analyses of glutamate receptor and its targets genes in myocardial and cerebral ischemia
Source: BMC Genomics. 2023 Jun 2;24:300. doi: 10.1186/s12864-023-09408-z (PMC10239159; doi:10.1186/s12864-023-09408-z)
Supplement: Supplementary file 1 — Supplementary Material 1 [file 12864_2023_9408_MOESM1_ESM.docx]

Additional file 1：

Immunofluorescence negative control of PC12 cells and H9c2 cells control group and model group


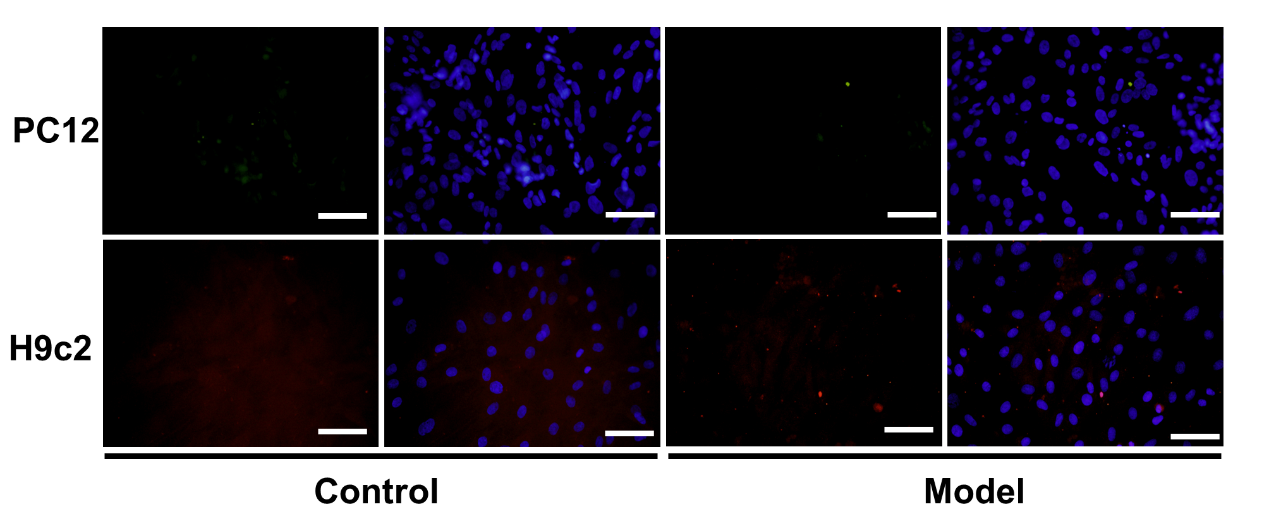
 Fig.S1. In the control and model groups of PC12 cells, negative controls indicate cells stained with control IgG-FITC. In the control and model groups of H9c2 cells, negative controls indicate cells stained with control IgG-Alexa (Bar=50μm).
